# Cultural and Institutional Issues in ACP for Care Home Residents: Perspectives of Loved Ones

**DOI:** 10.1093/geroni/igaa057.2717

**Published:** 2020-12-16

**Authors:** Brian de Vries, Gloria Gutman, Helen Kwan, Katrina Jang, Shimae Soheilipour, Avantika Vashisht, Taranjot Kaur

**Affiliations:** 1 San Francisco State University, San Francisco, California, United States; 2 Simon Fraser University, Vancouver, British Columbia, Canada; 3 Gerontology Research Centre, Vancouver, British Columbia, Canada; 4 Simon Fraser University Gerontology Research Centre, Vancouver, British Columbia, Canada

## Abstract

Focus groups were held with family/decision makers of residents in an exclusively Chinese (EC; N=7) and a multi-ethnic (ME; N=8) care home, as well as South Asian (SA; n = 5) and lesbian, gay, bisexual and transgender caregivers (LGBT; n = 5) who had/have a loved one in a care home. Shared themes across groups included the role of the care home in Advance Care Planning (ACP) discussions, the timing of such discussions (i.e., at admission), and the extent to which another person was available and appropriate for such discussions. Issues unique to groups included superstition and the equation of ACP with funeral planning (EC), family history and regrets about not having planned (ME), gender differences and the need for education about ACP (SA) and the absence of traditional family among LGBT older adults. These themes highlight the challenges in ACP among diverse populations and the need for targeted interventions.

